# Wearable Personal Uroflowmeter for Measuring Urine Leakage in Women with Incontinence: Feasibility Study

**DOI:** 10.3390/bios15080481

**Published:** 2025-07-24

**Authors:** Ali Attari, Faezeh Shanehsazzadeh, Tana Kirkbride, Carol Day, John O. L. DeLancey, James A. Ashton-Miller

**Affiliations:** 1 Department of Mechanical Engineering, University of Michigan, Ann Arbor, MI 48109, USA; faezehs@umich.edu (F.S.); jaam@umich.edu (J.A.A.-M.); 2 Procter & Gamble, Cincinnati, OH 45202, USA; kirkbride.tm@pg.com (T.K.); day.ca@pg.com (C.D.); 3 Department of Obstetrics & Gynecology, University of Michigan, Ann Arbor, MI 48109, USA; delancey@med.umich.edu; 4 Department of Biomedical Engineering, University of Michigan, Ann Arbor, MI 48109, USA; 5 Institute of Gerontology, University of Michigan, Ann Arbor, MI 48109, USA

**Keywords:** incontinence, uroflowmetry, urine leakage, flow rate sensor, wearable technology

## Abstract

This paper describes a novel wearable personal uroflowmeter and its use to log urine leakage episodes in women. Consisting of a miniature flow rate sensor attached under the urethral meatus, it recorded both urine flow rate and volume during activities of daily living. The sensor communicated with a determining unit incorporating a microcontroller and an inertial measurement unit worn at the waist, facilitating the post-hoc determination of which activities and changes in pose caused leakage. Six women participated in a feasibility study performed in a clinical setting. The results indicate that the uroflowmeter was 97.5% accurate in assessing micturition flow compared to gold standard uroflowmetry and leakage measurements. The system also provides subject-specific information on the relationship between physical activity and urine leakage, thereby eliminating errors due to missing data and recall bias in bladder leakage diaries and circumventing the limitations of office-based uroflowmeters.

## 1. Introduction

Lower urinary tract symptoms include urinary incontinence (UI), which is defined as the involuntary leakage of urine as a result of stress, urge, or mixed subtypes [1,2,3]. UI affects women globally, affecting over 60% at some point in their lives [4,5,6], diminishing the quality of life at a considerable cost [1,2,3,7,8,9]. The most common type of UI is stress urinary incontinence (SUI), which involves involuntary urine leakage during dynamic physical activities that include lifting, jumping, coughing, sneezing, and laughing [9,10]. Only 25% of affected women seek medical care, and less than half receive specialized treatment [10,11]. Barriers include a lack of awareness about treatments for SUI and the condition not being understood [10,12]. Insights into the triggers causing frequency and volume of urine leakage can help reveal causes, which in turn help clinicians customize treatment. Additionally, manufacturers of anti-incontinence products are interested in quantifying the volume and frequency of leakage in order to optimize product design.

A number of techniques have been developed to measure urine flow rate and voided volume. However, the accuracy of these can be compromised by disparities between the clinic and home environments. For example, artificial clinical conditions, pre-hydration, and a lack of privacy can affect the results. Uroflowmetry is a traditional clinical method for evaluating UI, measuring voiding urine flow rate and time to void, but offers little or no insight into the causes of incontinence that occur during free-ranging physical activities at work or home. While instrumented toilets, portable uroflowmeters, and handheld devices have been introduced to provide greater flexibility, they frequently encounter challenges related to inaccuracy, movement sensitivity, and reliability issues. Additionally, factors such as urine salinity can also affect device performance [13,14,15]. Home-based devices have emerged as alternatives, yet they struggle to gain widespread adoption due to functional, complexity, and cost issues. This underscores the need for practical, affordable, and easy-to-use home systems for the consistent evaluation of lower urinary tract symptoms. Existing non-invasive tools, tailored for laboratory use, fall short in reflecting real-life scenarios and are inadequate for tracking incontinence during activities of daily living (ADLs) [16,17,18].

A common clinical practice involves asking patients to maintain a leakage (or “bladder”) diary to evaluate urinary incontinence by recording the frequency and severity of leakage episodes over 3–7 days. However, these records are subjective in nature and their accuracy is marred by recall bias and the possibility that patients might not always feel or notice leakage episodes; furthermore, accurately estimating the volume of leaked urine is challenging. They are particularly impractical for older or cognitively impaired individuals [19,20,21,22]. Other objective methods to quantify urine loss include weighing absorbent pads [23], using wetness sensor wearable devices [24,25,26,27,28,29,30,31,32], employing urine collection bags [33], or performing the paper towel test held under the perineum [24]. However, these methods fail to measure the instantaneous flow rate and the number of leakage episodes during ADLs, and again, they are not useful for cognitively impaired or unresponsive individuals. Consequently, these limitations highlight a substantial gap in the development of more practical, effective incontinence measurement devices for everyday use.

In this paper, a novel wearable personal uroflowmeter (PUF) [34] is described. It was developed to improve the quantification of urinary incontinence during ADL. The PUF is designed to attach between the labia under the urethral meatus in women, capturing urine flow rate and leakage data during ADL-related leakage episodes. It also features an integrated inertial measurement unit (IMU) that records physical activity and body posture in real-time. Correlating the leakage events with the measured physical activity offers valuable insights into the specific activities that can trigger leakage episodes.

We describe the first-in-human testing of the PUF involving six women in a clinical setting during micturition, demonstrating the feasibility of wearable uroflowmetry. Initial results prompted several enhancements to improve the device’s performance and accuracy.

## 2. Materials and Methods

### 2.1. Ethics Statement

Testing sessions were carried out at one of the FemCare research facilities in a Procter & Gamble (Cincinnati, OH, USA) research complex under a UM IRB exemption (HUM00135969 and HUM00142989). The P&G safety committee for the P&G research facility also approved the device and the experiments described in this paper.

### 2.2. Experimental Design

This study was a single-center, cross-sectional, observational cohort study designed to quantify micturition and urinary incontinence leakage during different activities of daily living using the novel wearable personal uroflowmeter. The primary objective was to evaluate the PUF’s performance in measuring urine volume and flow rate during micturition and compare its data with standard scale-based uroflowmetry and pad weighing methods.

### 2.3. Study Population

Six women with urinary incontinence participated in this study. The participants had a mean (±standard deviation) age of 50 (±24) years and a mean BMI of 29.6 (±10) kg/m^2^. Participants were recruited by Procter & Gamble (P&G) from an internal pool of test participants.

### 2.4. Apparatus

**Figure 1:**
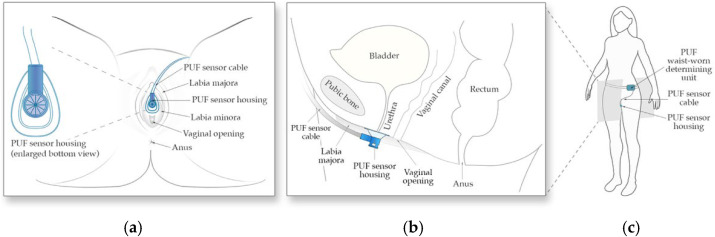
Schematic of the PUF apparatus. (**a**) Positioning of the wearable uroflowmeter under the urethral meatus in a female in lithotomy position; (**b**) A left lateral schematic representation of the PUF placement under the urethral meatus; (**c**) The apparatus worn by the subject.

**Figure 2:**
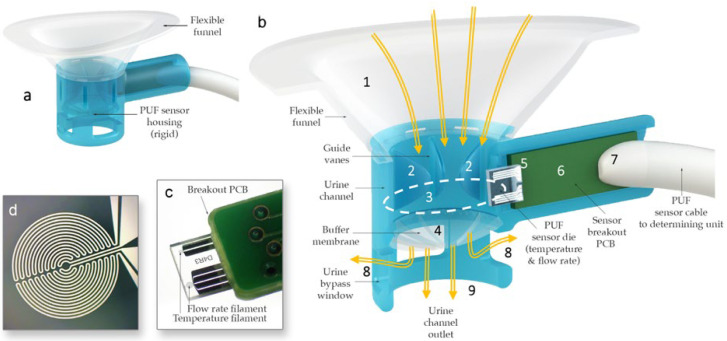
(**a**) PUF flowrate sensor housing. (**b**) Cross-sectional view of the PUF showing urine (yellow arrows) captured by the biocompatible flexible silicone funnel (1) passing through the guide vanes in the upper urine channel (2), the sensor channel internal aperture (dotted line) (3), and the post sensor convex flexible buffer membrane (4) in the lower urine channel; the glass substrate of the flow rate sensor (5) protrudes into the measurement channel to measure the instantaneous flow rate of the urine passing through the aperture; the substrate is mounted (wire bonded and encapsulated) onto a custom-made printed circuit board (6), which itself is soldered to a premium grade flexible cable (7); lateral bypass windows (8) are designed to allow urine to flow in the case that the main outlet (9) is obstructed by an incontinence pad. (**c**) Sensor die mounted on a PCB. (**d**) Magnified image of one of the anemometry filaments deposited.

The PUF consists of two main components, (1) the flow rate sensor and its funnel-shaped housing positioned under the urethral meatus between the labia to collect urine (Figure 1a,b), and (2) the determining unit worn at the waist which powers the sensor, records sensor readings, and tracks body movement data using an embedded 6-axis inertial measurement unit (Figure 1c).

The sensor housing (Figure 2) consists of a rigid urine measurement channel with a 10 mm diameter circular internal cross-section, which was rapid-prototyped using a Form 2 3D printer (Formlabs, Somerville, MA, USA). To ensure a comfortable fit across various labial shapes, a flexible funnel portion was over-molded onto the rigid channel using flexible medical-grade silicone elastomer MED2-4220 (Nusil Technology LLC, Carpinteria, CA, USA). Urine collected in the funnel passes through the measurement channel that quantifies flow rate and temperature using hotwire anemometry principles. Hot-wire anemometers operate by sensing how much a heated filament is cooled by the fluid flowing over it by keeping track of its electrical resistance. The sensor, adapted from the design by Lin et al. [35], was modified to measure a flow rate range from a single droplet to 40 mL/s. The PUF sensor die contains two filaments with equal resistance, each sensitive to both the temperature and flow rate of the fluid passing over them. However, the filaments are operated at different input power levels so that one becomes primarily sensitive to flow rate—though still affected by temperature—while the other serves as a temperature reference [35,36]. By leveraging the outputs from these two filaments, both flow rate and temperature can be measured.

Additionally, the PUF sensor housing incorporates guide vanes within the urine flow channel to minimize swirl and enhance flow rate measurement accuracy, an ultra-flexible membrane to maintain consistent flow at lower rates by keeping the sensor aperture primed, and side vents to allow urine to escape if the main outlet becomes obstructed by the incontinence pad. Finally, the sensor was positioned at an approximately 11-degree incline relative to the flow direction in the urine channel to prevent flow separation from the filaments, thereby ensuring more precise measurements.

The PUF’s determining unit was comprised of four AAAA-size, 1.5 V batteries, a power regulation and constant current sources, an amplifier section, a microcontroller (AdaFruit ESP32 Feather Board (Adafruit Industries, Industry City, Brooklyn, New York, NY, USA), SD memory with 32 GB capacity, and an inertial measurement unit (NXP, FXOS8700 accelerometer and magnetometer (NXP Semiconductors, Eindhoven, The Netherlands), FXAS21002 gyroscope (NXP Semiconductors, Eindhoven, The Netherlands)). The latter was mounted in the waist-worn determining unit to record body posture and movement data simultaneously with leakage and flowmetry data. Data was acquired by the microcontroller at a 40 Hz sampling rate. During post-processing, signals were regulated to 20 Hz and filtered using a third-order lowpass (Butterworth) filter with a 2 Hz cutoff frequency.

### 2.5. Measurement Reference and Sensor Calibration

**Figure 3:**
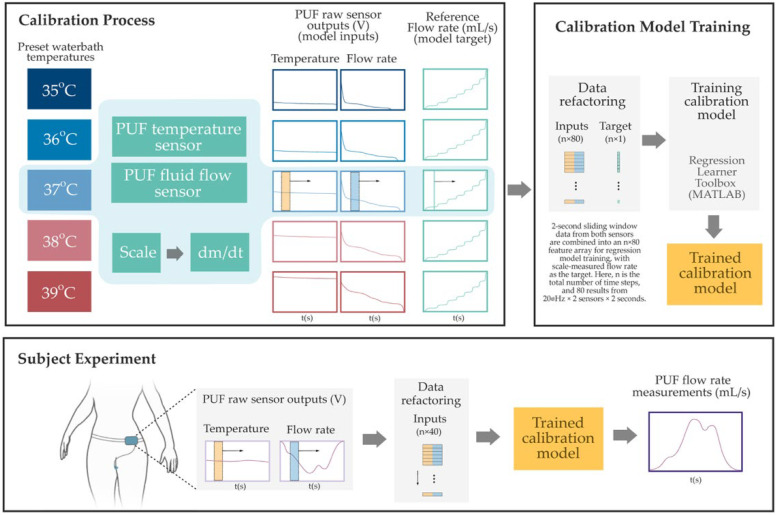
Overview of the PUF flow rate sensor calibration and estimation process. The Top Left Panel shows the benchtop calibration setup, where raw voltage signals from the PUF flow and temperature sensors are collected under controlled water bath temperatures while the flow rate is gradually increased from 0 to 40 mL/s. Reference flow rates are derived from time-differentiated scale measurements. The Top Right Panel illustrates the data refactoring process, two-second sliding windows—highlighted by semi-transparent yellow and blue rectangles in the left panel—are ex-tracted from both sensor signals and reshaped into an n × 80 input array for regression model training. The corresponding instantaneous reference flow rate, indicated by a vertical green line (also shown in the left panel), serves as the training target. Finally, in the Bottom Panel, the resulting sensor-specific model was used to estimate the instantaneous PUF flow rate obtained during subject experiments.

The PUF sensors were calibrated using a temperature-controlled water bath (PolyScience, Niles, IL, USA) and a high-precision scale (Sartorius ENTRIS6202-1S, Göttingen, Germany) as reference standards. The goal was to establish a mapping between the raw sensor outputs and known physical measurements. Heated distilled water was pumped through the sensor housing, and the scale continuously recorded the cumulative mass of the outflow. The reference flow rate was computed by time-differentiating the scale data, and the PUF simultaneously recorded flow and temperature signals. Calibration was conducted in 1 °C increments from 35 °C to 39 °C (±2 °C around body temperature). MATLAB R2024b’s Regression Learner Toolbox was used to create sensor-specific calibration models. As illustrated in Figure 3, input features were generated by applying a two-second sliding window (updated at every sample) over the temperature and fluid flow sensor output voltages. A regression model was then trained using the combined outputs of flow and temperature sensors. The model architecture consisted of three intermediate hidden layers with 128, 32, and 4 neurons, respectively, using sigmoid activations. Training included L2 regularization (Lambda = 1.5 × 10^−3^), input standardization, and a maximum of 1000 iterations. This structure was selected using an elbow-method-inspired approach, starting from a higher neuron count and progressively reducing layer sizes to balance accuracy and complexity. Holdout validation (50% data split) yielded a root mean square error (RMSE) around 2 mL/s. To simulate in vivo thermal conditions, water at the same temperature was circulated around the sensor housing during calibration. Repeatability tests on a subset of sensors at each fluid temperature confirmed stable performance, with minimal variation (RMSE < 1 mL/s).

### 2.6. Study Procedures

To ensure standardized conditions for urine flow measurement, participants followed specific preparation guidelines before their visits to the testing facility. They were instructed to drink two glasses of water and refrain from urinating for two hours prior to their arrival. Upon entering the facility, a registered nurse customized the perimeter of a hydrocolloid adhesive patch to attach the flexible funnel of the sensor housing to the body, ensuring a comfortable fit for each participant. This adhesive was designed to securely attach the sensor housing to mucosal and wet surfaces, and an extra-thin wound dressing layer was overlaid for additional support.

**Figure 4:**
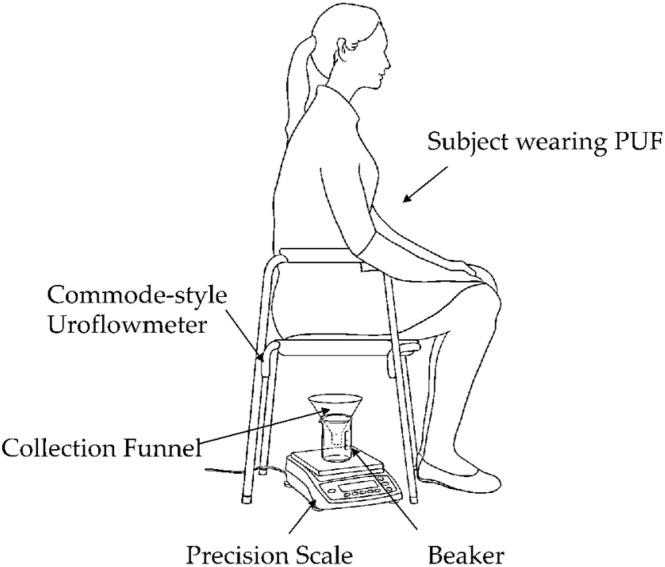
Commode-style uroflowmeter setup using a urine collection funnel and beaker placed on a precision scale. The weight read from the scale was sent to a computer to obtain the micturition flow rate at 20 Hz continuously throughout each micturition episode.

*Voiding test:* Each subject micturated in a private room while sitting on a commercial commode-style uroflowmeter with the PUF still attached. As shown in Figure 4, this setup included a commode-style uroflowmeter positioned above a urine collection container resting on a precision scale. The precision scale served as the reference uroflowmeter, measuring the urine’s weight over time to determine the flow rate.

*Longer-duration mobility tests:* Participants then wore pre-weighed absorbent pads under their clothing to quantify urine leakage over a specified duration. Additionally, they wore the PUF determining unit around their waist to monitor urine flow and physical activity synchronously.

Subjects performed a set of nine predefined ADLs while wearing the PUF, including walking, running on a treadmill, climbing stairs, bending over, squatting, lifting 1-gallon water bottles, lying on a bed, and sitting and rising from a chair. These activities were conducted to assess the PUF’s attachment strength and identify any specific activities that might lead to leakage.

After these activities, the nurse assessed the attachment of the PUF patch, removed the absorbent pad, weighed it, and noted the difference in weight as the reference for leakage volume.

Subjects repeated these steps up to three times for up to four hours. During each attachment examination, the nurse noted any areas where the patch had detached and finally removed the device from the subject.

After completing all experiments, the trained calibration model was employed to estimate the instantaneous PUF flow rate obtained during each experiment.

### 2.7. Statistical Analysis

The micturition flow rate profiles obtained from the PUF and the scale-based uroflowmeter were compared using the cross-correlation analysis [37].

To compare the leakage amount during ADLs, the difference in the absorbent pad weight before and after wear was compared with the cumulative measurements of PUF during the ADLs.

## 3. Results

**Figure 5:**
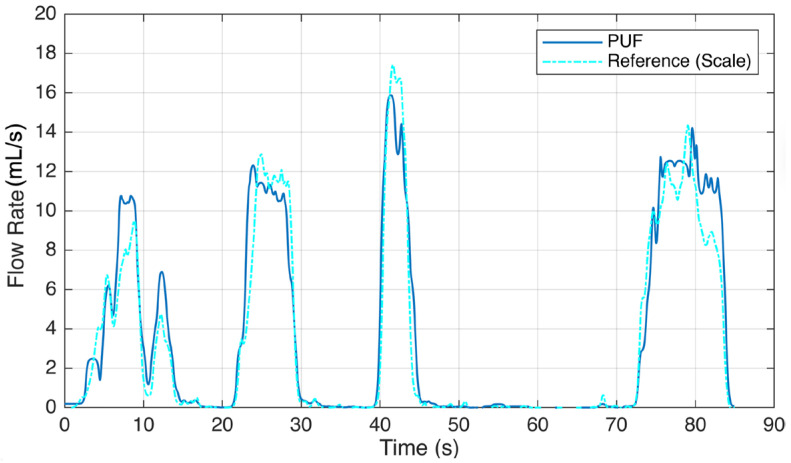
Voiding test result showing a sample urine flow rate recording of the PUF worn by a participant (Subject #5) trying to void. She voluntarily paused at 10, 15, 30, 46, and 85 s.

The results of this study reveal the performance and accuracy of the PUF device in both measuring urine flow during voiding tests and detecting leakage. In both types of experiments, each participant was asked to perform the tasks multiple times, with each repeated trial reported as an individual event. Six subjects experienced 16 voiding episodes while wearing the PUF. Figure 5 illustrates sample readings comparing the PUF device with the gold standard reference. Five episodes were excluded due to PUF patch detachment and bypass leakage, which caused inaccurate flow rate recordings. In the remaining 11 sessions, uroflowmetry profiles from the PUF closely matched those from the standard uroflowmeter, with a mean cross-correlation factor of 0.95 and an average RMSE (±2 SD) of 3.3 (±7.5) mL/s. By placing the PUF directly at the urethral meatus, we obtain immediate flow rate measurements, avoiding the temporal lag inherent to scale-based reference systems. This real-time responsiveness enhances the accuracy of the voiding pattern and timing characterization. To account for the delay in fluid reaching the collection container, we applied lag-adjusted cross-correlation to align the PUF and reference flow signals before computing the RMSE, ensuring an accurate comparison. The results are shown in Table 1.

Results from the second testing session with three subjects are summarized in Table 2, which compares the total voided urine volume recorded by the instrumented PUF with the pad weight gain after the experiment. The PUF was unable to measure leakage episodes for the remaining three subjects due to thermal drift in the sensor’s instrumentation circuit when the device was worn under winter clothing during high-intensity ADLs. This issue will subsequently be addressed in the next revision of the instrumentation circuit.

**Figure 6:**
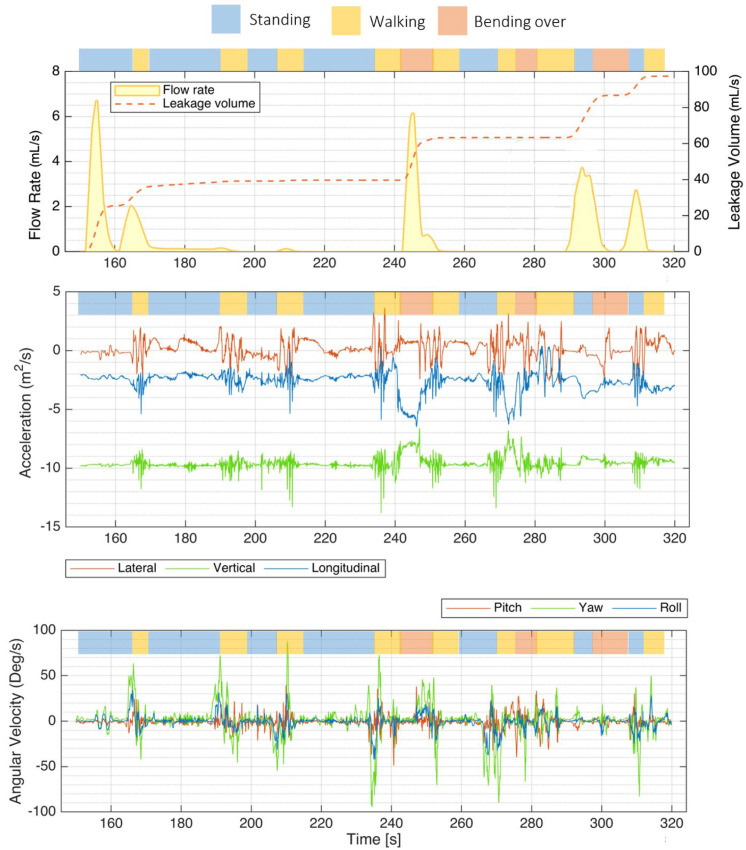
Mobility test results showing a sample flow rate, leakage volume, and IMU signals recorded by the PUF performed by one participant (S3). The top bar above each plot indicates the activity type—walking (yellow), standing (blue), and bending over (red). The top plot shows the flow rate and leakage volume recorded with the PUF. The middle plot presents the IMU accelerations along the three orthogonal axes, lateral, vertical, and longitudinal. The bottom plot illustrates rotational rates around the transverse (lateral, red), vertical/yaw (green), and longitudinal/roll (blue) axes, with green spikes indicating moments when the subject turned on her feet.

As illustrated in Figure 6, the instrumented PUF unit effectively recorded sample flow rate, leakage volume, and IMU signals during various activities performed by a panelist at the P&G testing facility (S3). Key activities included “walking” (highlighted in yellow), “standing” (blue), and “bending over” (red).

Accelerations along each of the three orthogonal axes were the most helpful components of the IMU signals for identifying posture and activity type. The projection of the gravity vector on each axis was mainly used to identify posture.

The bottom plot illustrates rotational rates around different axes: the transverse (lateral) axis in red, the vertical (yaw) axis in green, and the longitudinal (roll) axis in blue. Notably, green spikes in the yaw signal correspond to moments when the subject turned on her feet.

## 4. Discussion

The adhesive layers and design of the PUF enabled secure attachment to the mucosal surface between the labia for up to four hours during daily activities and across multiple voiding episodes. This duration allowed for repeated mobility testing within a clinically relevant timeframe. While the system is technically capable of longer-term signal monitoring, clinical wear time was primarily limited by how long secure adhesion of the sensor to the body can be maintained. However, we found that effective adhesion required prior cleaning and drying of the application site. Flow rates exceeding 40 mL/s occasionally caused a detachment of the device, posing challenges for measurement in patients with urge urinary incontinence. This could be addressed in the future by employing a larger-diameter urine channel in those women.

Despite only leakage measurements on three subjects, this study demonstrated the feasibility of using wearable uroflowmetry to monitor leakage episodes in women with SUI. The remaining recordings were corrupted due to sensor housing detachment or thermal drift in the determining unit. Benchtop studies without subjects confirmed that once the channel was primed, the sensor could measure flow rates from a single droplet to 40 mL/s. Saturation occurred at flow rates above 40 mL/s, indicating the need for further refinement, but such high flow rates may not be common or usual. The sensor’s output voltage was temperature-dependent, posing challenges. While the adopted calibration method was sufficiently accurate for steady-state flow (e.g., micturition), its performance was reduced during transient flow events (e.g., low-volume leakage), indicating the need for future calibration refinement.

Experimental flowmetry data showed that in the voiding tests, the mean PUF measurement error was 2.5% when compared to the reference values obtained using the gold standard clinical scale-based measurement, as reported in Table 1. In the mobility tests, the overall accuracy was calculated as 60.6%, based on the average of absolute percentage errors across three representative use cases. In all studies, accuracy was defined as 100% minus the mean of absolute errors, to avoid the cancellation that occurs when signed errors (i.e., both positive and negative) are averaged. Using absolute values ensures a more accurate reflection of the true magnitude of deviation between the PUF measurements and the reference method. Reasons for this moderate accuracy are discussed under Limitations (below).

A key advantage of this uroflowmeter over traditional clinical devices is its ability to monitor body posture and movements associated with leakage. Compared to wetness sensors, the PUF stands out by measuring instantaneous flow rate, total urine leakage volume, and providing data on the pose of the subject, offering comprehensive insights into urinary incontinence patterns. The accelerometer data were particularly effective for identifying posture and activity types, because periodic oscillations in vertical and longitudinal accelerations were indicative of dynamic activities like walking and running.

### 4.1. Limitations

One limitation of this study was the lack of any other instantaneous gold standard measurement system to quantify urine leakage measured by PUF. Although the pad weight difference method served as the gold standard, it only provided cumulative data, which prevented a real-time, direct comparison with the sensor data. Moreover, one potential source of measurement inaccuracy between the urine micturition volume measured by a precision scale and that obtained from the sensor is likely due to hydrodynamic modifications to flow caused by the sensor housing and its membranes. This discrepancy may explain the differences observed in the maximum flow rates recorded by the PUF compared to those measured by the scale.

Apart from the limitations of the reference measurement systems, the PUF also exhibited certain inherent limitations. The urine channel housing opening was sufficiently large (10 mm in diameter) to facilitate unobstructed micturition; however, this design was potentially sub-optimal for measuring individual droplets because the channel could not consistently be primed with urine. In such cases, specific postures caused droplets to bypass the sensor filaments, while high-acceleration activities led to droplets repeatedly passing over the sensor without exiting the urine channel, resulting in phantom measurements. The latter phenomenon was further exacerbated when small droplets adhered to slight irregularities on the surface of the urine channel. Future designs could address the latter issue through the application of a hydrophobic surface treatment. Finally, software-level compensation could help mitigate the phantom measurements by utilizing the data already recorded by the inertial sensors integrated in the PUF.

Thermal inertia affects the system’s responsiveness and accuracy in measuring flow rate changes. Machine learning techniques were employed, using historical data from the past two seconds, to predict actual flow rates and alleviate this issue.

Temperature drift in the determining unit circuit emerged as a critical source of error in the PUF device, particularly when panelists tested the device under winter clothing for periods longer than an hour or during high-intensity ADLs, which resulted in a systematic rise in body temperature. For example, in one case, when the determining unit was worn outdoors under warm winter clothing, the onboard temperature sensor of the determining unit increased from 21 °C to 41 °C. This temperature rise caused the sensor’s calibration settings to drift within 20 min, leading to signal saturation due to the sensitivity of certain electrical components to temperature changes. The addition of an effective temperature compensation circuit could address this issue in the future.

Another issue was that the system output varied between 2.8 and 3.2 V, depending on sensor impedance. In earlier designs, manual calibration was performed using an MCP47FEB22 (Microchip Technology Inc., Chandler, AZ, USA) digital-to-analog converter (DAC) from Microchip Technology to adjust the DC output level and accommodate a wide range of flow rates. Despite this, the DC levels fluctuated with each activation, necessitating frequent and time-consuming recalibration. These issues collectively contributed to inaccuracies in flow rate measurement and indicated the need for an improved instrumentation circuit design.

Finally, since this was a feasibility study, the subject pool was limited. In addition to increasing the number of participants with stress urinary incontinence, it will be important to recruit individuals with urge incontinence to assess the PUF funnel’s ability to remain attached during high-flow voids or urge incontinence episodes. These scenarios can potentially lead to PUF detachment or bypass leakage, thereby compromising measurement accuracy. If these should occur, one could use a larger diameter measurement channel.

### 4.2. System Improvement After Subject Testing

To address the critical issues of (1) temperature drift, (2) DC voltage drift, and (3) low dynamic range, we redesigned the analog sensor interface of the PUF device. Our approach involved eliminating the DC offset from the sensor output. We then implemented a two-stage low-noise amplifier configuration to differentially amplify the signal. Finally, we adjusted the DC level to meet the specifications of a single supply circuit, ensuring optimal performance.

**Figure 7:**
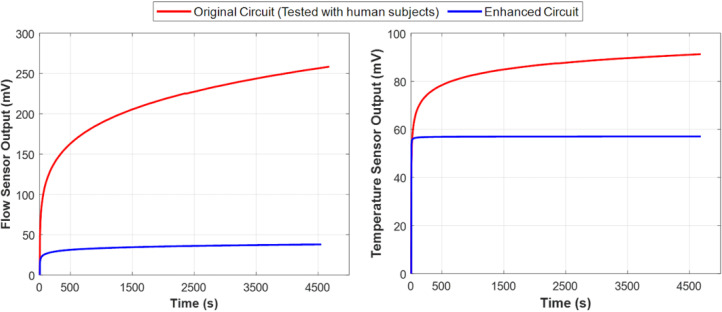
Comparison of performance (voltage drift) between the original instrumentation circuit used in the experiment (red) and the enhanced design (blue) with respect to temperature rise. These plots demonstrate that despite the inevitable temperature rise in the determining unit circuit, the enhanced instrumentation circuit performed more stably and exhibited significantly less output voltage drift over time, as shown in both flow rate sensor measurements (**left**) and urine temperature sensor measurements (**right**). Both sensor outputs are essential for accurately measuring the true urine flow rate.

Figure 7 compares the performance of the original and the enhanced circuit designs by depicting the amplified output voltage of the flow rate sensor and the temperature sensor over time in the absence of fluid flow. The results indicate that the new design stabilized within eight minutes and remained stable even as the circuit temperature increased to 32 °C. These findings confirm that the first and second issues have been effectively addressed.

Moreover, the output is now independent of the DC offset caused by sensor impedance, allowing for adjustable gain across all sensors. This enhancement increases resolution and accuracy without data loss, effectively resolving the third issue. In subsequent measurements, the system operated without saturation.

## 5. Conclusions

This development and first-in-human testing of a novel miniature wearable personal uroflowmeter shows that it is feasible to objectively measure urine flow rates and volumes in women with urinary incontinence during their daily activities, an impossibility with current incontinence assessment methods. In tests involving six incontinent women, the PUF demonstrated high accuracy for micturition flow measurements. Even with a moderate accuracy of 60.6% in quantifying ADL-related leakage, it still offers valuable insights into the relationship between physical activities and leakage episodes. We identified that improvement was needed for sensor saturation and temperature drift, which we later showed was remediable. Although the tests were conducted on women, the system is easily adapted for measuring incontinence in men by attaching it to the end of a condom. Future work will focus on improving sensor robustness for longer-term monitoring, enhancing leakage detection accuracy through advanced algorithms, and expanding clinical trials to larger and more diverse patient populations to validate and optimize real-world performance.

## 6. Patents

The PUF is described in US patent 0121112A1, 2021 [34].

## Figures and Tables

**Table 1:** Voiding results showing a summary of the recordings of the PUF device compared with the reference uroflowmeter in 11 voiding episodes.

	Cross Correlation	Micturition Volume (mL)			Flow Rate RMSE (mL/s)	Maximum Flow Rate (mL/s)	
		Reference	Measured (PUF)	Measurement Error (%)		Reference	Measured (PUF)
S1-V1 *	0.95	239.0	226.1	5.4	12.9	18.8	17.1
S1-V2	0.90	354.7	347.7	2.0	7.0	30.2	26.4
S2-V1	0.97	86.7	87.3	0.7	0.6	11.4	11.3
S2-V2	0.98	70.5	71.6	1.6	1.1	8.8	8.4
S3-V1	0.98	118.7	120.1	1.2	1.5	15.8	15.9
S4-V1	0.96	193.2	193.9	0.4	0.7	15.2	15.9
S4-V2	0.99	294.5	291.7	0.9	2.8	25.2	21.8
S5-V1	0.94	39.3	41.4	5.2	2.0	9.0	8.0
S5-V2	0.96	120.3	120.4	0.1	0.1	28.1	25.0
S6-V1	0.94	86.7	90.0	3.8	3.3	11.4	13.1
S6-V2	0.93	70.8	75.2	6.2	4.4	38.3	33.7

* S: Subject Number, V: Voiding Event Number.

**Table 2:** Mobility results showing a comparison of total voided urine volume recorded by the instrumented PUF with pad weight gain in three subjects during the second testing session.

	Reference Leakage Amount (mL)—Pad	Measured Leakage Volume (mL)—PUF	Volume Difference (mL)	Percent Error (%)
S3	101.8	164.6	62.8	61.7
S4	37.9	24.1	13.9	36.6
S6	35.6	28.5	7.0	19.8

## Data Availability

The data will be made available upon request from the corresponding author.

## References

[1] Frigerio M., Barba M., Cola A., Braga A., Celardo A., Munno G.M., Schettino M.T., Vagnetti P., De Simone F., Di Lucia A. (2022). Quality of Life, Psychological Wellbeing, and Sexuality in Women with Urinary Incontinence-Where Are We Now: A Narrative Review. Medicina.

[2] McKinney J.L., Datar M., Pan L.C., Goss T., Keyser L.E., Pulliam S.J. (2022). Retrospective Claims Analysis of Physical Therapy Utilization among Women with Stress or Mixed Urinary Incontinence. Neurourol. Urodyn..

[3] Dasdelen M.F., Almas F., Celik S., Celik N., Seyhan Z., Laguna P., Albayrak S., Horuz R., Kocak M., Rosette J. (2023). When Bladder and Brain Collide: Is There a Gender Difference in the Relationship between Urinary Incontinence, Chronic Depression, and Anxiety?. J. Clin. Med..

[4] Gutiérrez V.B., Hundley V.A., Way S. (2023). The Experience of Women From Underrepresented Groups With Urinary Incontinence: A Systematic Review. J. Transcult. Nurs..

[5] Swanton A.R., Gormley E.A. (2020). Prevention of Urinary Incontinence in Women. Curr. Urol. Rep..

[6] Vaughan C.P., Markland A.D. (2020). Urinary Incontinence in Women. Ann. Intern. Med..

[7] Moossdorff-Steinhauser H.F.A., Berghmans B.C.M., Spaanderman M.E.A., Bols E.M.J. (2021). Prevalence, Incidence and Bothersomeness of Urinary Incontinence in Pregnancy: A Systematic Review and Meta-Analysis. Int. Urogynecol. J..

[8] Datar M., Pan L.C., McKinney J.L., Goss T.F., Pulliam S.J. (2022). Healthcare Resource Use and Cost Burden of Urinary Incontinence to United States Payers. Neurourol. Urodyn..

[9] Lugo T., Leslie S.W., Mikes B.A., Riggs J. (2024). Stress Urinary Incontinence.

[10] Moris L., Heesakkers J., Nitti V., O’Connell H.E., Peyronnet B., Serati M., Omar I.M., Harding C. (2025). Prevalence, Diagnosis, and Management of Stress Urinary Incontinence in Women: A Collaborative Review. Eur. Urol..

[11] Sutcliffe S., Falke C., Fok C.S., Griffith J.W., Harlow B.L., Kenton K.A., Lewis C.E., Low L.K., Lowder J.L., Lukacz E.S. (2024). Lower Urinary Tract Symptoms in US Women: Contemporary Prevalence Estimates from the RISE FOR HEALTH Study. J. Urol..

[12] Li Q., Cheng Y., Shi H., Xue K., Zhou F. (2023). Advances in the Natural History of Urinary Incontinence in Adult Females. J. Obstet. Gynaecol..

[13] DiMino A., Drummer M.E., Campbell J.M., Malik A., Curameng L. (2017). Apparatus and Method for Uroflowmetry. U.S. Patent.

[14] Beckwith R., Drinnan M., Bray A., Griffiths C.J., Whitaker M. (2016). Urine Flow Measuring Apparatus. E.P. Patent.

[15] Ams F., Baier M., Schäfer R. (1995). Flow Measurement Means of Body Liquid. E.U Patent.

[16] Gammie A., Wachter S.D. (2024). Research Priorities for Diagnostic Instrumentation in Urinary Incontinence. Proc. Inst. Mech. Eng. H.

[17] Dawidek M.T., Singla R., Spooner L., Ho L., Nguan C. (2022). Clinical Validation of an Audio-Based Uroflowmetry Application in Adult Males. Can. Urol. Assoc. J..

[18] Chun K., Kim S.J., Cho S.T. (2017). Noninvasive Medical Tools for Evaluating Voiding Pattern in Real Life. Int. Neurourol. J..

[19] Lukacz E.S., Santiago-Lastra Y., Albo M.E., Brubaker L. (2017). Urinary Incontinence in Women: A Review. JAMA.

[20] Nygaard I., Holcomb R. (2000). Reproducibility of the Seven-Day Voiding Diary in Women with Stress Urinary Incontinence. Int. Urogynecol. J. Pelvic Floor. Dysfunct..

[21] Tincello D.G., Williams K.S., Joshi M., Assassa R.P., Abrams K.R. (2007). Urinary Diaries: A Comparison of Data Collected for Three Days versus Seven Days. Obstet. Gynecol..

[22] Rabin J.M., McNett J., Badlani G.H. (1993). Computerized Voiding Diary. Neurourol. Urodyn..

[23] Ryhammer A.M., Djurhuus J.C., Laurberg S. (1999). Pad Testing in Incontinent Women: A Review. Int. Urogynecol J. Pelvic Floor. Dysfunct..

[24] Miller J.M., Ashton-Miller J.A., Delancey J.O.L. (1998). Quantification of Cough-Related Urine Loss Using the Paper Towel Test. Obstet. Gynecol..

[25] Li E. (2019). Intelligent Incontinence Monitor Generating and Utilizing Incontinence Profiles. U.S. Patent.

[26] Curran P., Barda D.A., Bradley D.B., Phillips J., Kotlarski P., Olkkonen J.T., Juha T., Mattila P. (2016). Capacitive Wetness Sensor and Method for Manufacturing the Same. E.P. Patent.

[27] Lewis P.M., Carey K.M., Templestowe L., Cottenden A.M., Barda D.A., Curran P., Black D. (2018). Incontinence Monitoring and Assessment. U.S. Patent.

[28] Ang L.M., Ow S.H., Seng K.P., Tee Z.H., Lee B.W., Thong M.K., Poi P.J.H., Kunanayagam S. (2008). Wireless Intelligent Incontinence Management System Using Smart Diapers. Proceedings of the 2008 5th International Conference on Electrical Engineering/Electronics, Computer, Telecommunications and Information Technology.

[29] Flack F.C., James E.D. (1973). Incontinence Measurement Sensor. U.S. Patent.

[30] Ramirez F. (2014). Incontinence Detection System. U.S. Patent.

[31] Addington W.R., Miller S., Stephens R.E. (2014). Techniques for Evaluating Urinary Stress Incontinence. U.S. Patent.

[32] Dick B.R., Duke R.T., Osborne E.E., Sable S.P., Scott T.E., Taverner C. (1996). Incontinence Detection Device. U.S. Patent.

[33] Johnson D.E., Muncie H.L., O’Reilly J.L., Warren J.W. (1990). An External Urine Collection Device for Incontinent Women. Evaluation of Long-Term Use. J. Am. Geriatr. Soc..

[34] Attari A., Ashton-Miller J.A., Delancey J.O., Burns M.A., Kirkbride T.M., Carlin E.P., Ramachandran A.J., Day C.A. (2025). Uroflowmetry Systems Having Wearable Uroflowmeters, and Methods of Operating the Same, and Methods of Operating the Same. U.S. Patent.

[35] Lin W.C., Burns M.A. (2015). Low-Power Micro-Fabricated Liquid Flow-Rate Sensor. Anal. Meth..

[36] Shanehsazzadeh F., DeLancey J.O.L., Ashton-Miller J.A. (2025). Improving the Accuracy of a Wearable Uroflowmeter for Incontinence Monitoring Under Dynamic Conditions: Leveraging Machine Learning Methods. Biosensors.

[37] Bendat J.S., Piersol A.G. (2012). Random Data: Analysis and Measurement Procedures.

